# A concise guide to fluorescent cell cycle reporters for live-cell imaging

**DOI:** 10.3389/fcell.2025.1702230

**Published:** 2025-10-20

**Authors:** Jinyu Wang, Yige Li, Jia Luo, Septavera Sharvia, Kuan Yoow Chan

**Affiliations:** ^1^ Centre for Cellular Biology and Signalling, Zhejiang University-University of Edinburgh Institute, Zhejiang University School of Medicine, Zhejiang University, Haining, China; ^2^ Faculty of AI, University Teknologi Malaysia, Kuala Lumpur, Malaysia; ^3^ Department of Biomedical Sciences, University of Birmingham, Birmingham, United Kingdom

**Keywords:** cell cycle, FUCCI, HDHB, KTR, PCNA

## Abstract

The cell cycle is a fundamental process vital for organismal growth and stability. Its dysregulation underlies many human diseases, in particular cancers, making its monitoring essential in biological research. Genetically encoded fluorescent cell cycle reporters have become indispensable tools for studying the cell cycle, providing invaluable insights into cell cycle dynamics at single-cell resolution. A variety of fluorescent reporters, including FUCCI, kinase translocation reporters, and DNA replication foci-based systems, have been developed to track cell cycle progression. Each reporter measures distinct cell cycle specific processes to determine the cell cycle status, exhibiting distinctive strengths and limitations. In this review, we provide an overview on the commonly used cell cycle reporter systems. We then highlight the strengths and weaknesses of the various cell cycle reporter systems to guide researchers in selecting the most appropriate reporters for their specific needs. Finally, we discuss recent approaches where various cell cycle reporters are combined to overcome the limitations of each system. Collectively, single cell analysis with these reporters are transforming the study of cell cycle regulation, advancing our ability to interrogate a fundamental process that governs cell fate and function.

## Introduction

The cell cycle is an important biological process that ensures the accurate replication and division of genetic material into two daughter cells (Morgan, 2007). This process is essential for development, tissue repair, and maintaining stability within organisms. Faults in cell cycle regulation are associated with various human diseases, including congenital disorders and cancer (Joseph et al., 2020; Matthews et al., 2021). Consequently, monitoring the cell cycle is crucial for many biological studies.

Various methods have been developed to perform cell cycle studies. One of the most popular is the use of analytical flow cytometry to assess cell cycle phase distribution in a heterogeneous cell population. This technique relies on DNA stains, sometimes combined with antibody markers of cell cycle proteins, to determine the cell cycle status of a population of cells (Darzynkiewicz et al., 2010; Rieger, 2022). While highly informative, it requires harvesting cells at a single time point, offering a static snapshot of the cell cycle and lacking temporal resolution.

To gather more temporal data on cell cycle dynamics, drug-based cell cycle synchronisation methods such as the double thymidine block (Vogel et al., 1978; Chen and Deng, 2018), or with the CDK4/6 inhibitor Palbociclib (Trotter and Hagan, 2020; Chan et al., 2025), can be employed. While these techniques help track cell populations that progress synchronously through the cell cycle, the synchronisation methods themselves cause significant disturbances and cellular stress that can complicate biological interpretation (Gong et al., 1995; Kurose et al., 2006; Matson et al., 2019; Crozier et al., 2022). Additionally, the rapid loss of cell synchrony can conceal changes in cell cycle dynamics, further reducing the usefulness of these methods.

Consequently, methods capable of monitoring cell cycle status in real-time within single living cells have been developed. Experimental approaches using time-lapse imaging of actively proliferating cells have become increasingly popular for studying cell cycle dynamics (Muzzey and Van Oudenaarden, 2009; Skylaki et al., 2016; Cooper and Bakal, 2017). Advances in computational image analysis, especially in automated cell segmentation and lineage tracing, are enhancing the accessibility of microscopy-based cell cycle studies (Cheng et al., 2021; Midtvedt et al., 2021; Gui et al., 2022; Maška et al., 2023; Li et al., 2024). These methods frequently employ genetically encoded fluorescent cell cycle reporters to track cell cycle progression in live cells.

Various fluorescent cell cycle reporters have been developed to observe cell cycle progression. Notable examples include the Fluorescent Ubiquitination-based Cell Cycle Indicator (FUCCI) system (Sakaue-Sawano et al., 2008), kinase translocation reporters (Regot et al., 2014), and DNA replication foci–based reporters (Zerjatke et al., 2017). While these tools have significantly enhanced our ability to study cell cycle dynamics, their different mechanisms, readouts, and limitations mean that choosing the most appropriate reporter system depends heavily on the experimental context. As the number and complexity of available reporters grow, a careful assessment of their design principles, advantages, and disadvantages becomes increasingly important. This review aims to offer a comparative analysis of these fluorescent cell cycle reporters to help researchers select the most suitable options for their biological question.

## The FUCCI system

Among live cell reporters for cell cycle analysis, the Fluorescent Ubiquitination-based Cell Cycle Indicator (FUCCI) system stands out as the first genetically encoded fluorescent reporter used for the visualisation of cell cycle transitions. Introduced in 2008, FUCCI remains one of the most widely used genetically encoded systems for tracking cell cycle dynamics in both *in vitro* and *in vivo* settings (Sakaue-Sawano et al., 2008).

**FIGURE 1:**
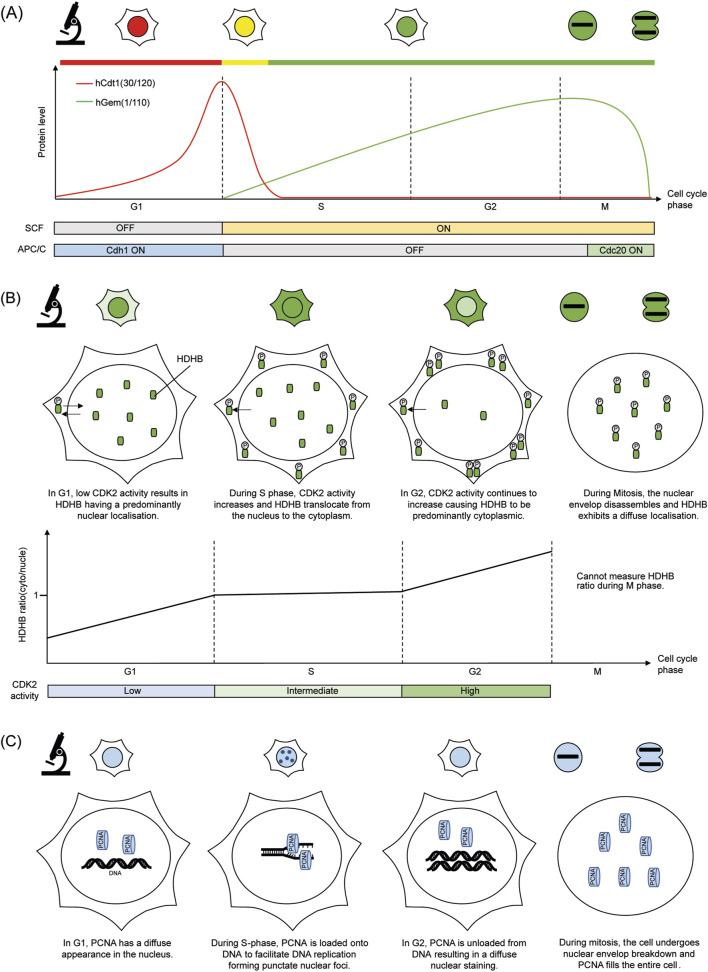
Simplified cartoon summarising the molecular basis of each cell cycle reporter and its fluorescence readout under the microscope. **(A)** FUCCI **(B)** HDHB KTR **(C)** PCNA based DNA replication foci.

The FUCCI system relies on the cell cycle-controlled breakdown of two essential proteins: Cdt1 and Geminin (Sakaue-Sawano et al., 2008). Cdt1 is involved in the regulation of DNA replication (Nishitani et al., 2000), while Geminin is an inhibitor for DNA replication in cells (McGarry and Kirschner, 1998). These proteins are targeted for destruction by specific E3 ubiquitin ligase complexes, APC^Cdh1^ and SCF^Skp2^, which become active during G1 and S/G2/M phases of the cell cycle respectively (Wohlschlegel et al., 2000; Nishitani et al., 2001; Li et al., 2003; Vodermaier, 2004). To act as phase-specific reporters, peptide sequences responsible for the cell cycle specific degradation of either Cdt1 or Geminin were fused with fluorescent proteins. In the original version of the FUCCI system, the reporter mKusabiraOrange2-hCdt1 (30/120) builds up in G1, while mAzamiGreen-hGem (1/110) accumulates in S/G2/M phases (Sakaue-Sawano et al., 2008). Their distinct degradation patterns result in red (G1), green (S/G2/M), or yellow at the G1/S transition due to overlapping signals (Figure 1A).

FUCCI depends on highly conserved post-translational regulation of the cell cycle through ubiquitin-mediated proteolysis. This feature allows FUCCI constructs to be expressed under various cell types while maintaining specificity (Sakaue-Sawano et al., 2008; Zielke and Edgar, 2015). It also facilitates stable and widespread expression in transgenic animals, making FUCCI suitable for long-term *in vivo* imaging (Mort et al., 2014; Zielke et al., 2014; Shcherbakova et al., 2016; Ford et al., 2018; Cura Costa et al., 2021; Hecht et al., 2022).

Since its introduction, FUCCI has been widely adopted to study various cell cycle dependent biological responses. Its ability to report cell cycle phase in real time and at single-cell resolution, has been particularly valuable in developmental biology, where spatial and temporal patterns of cell cycling influence organ formation, morphogenesis and repair (Sugiyama et al., 2009; Ogura et al., 2011; Calder et al., 2013; Lavado et al., 2018; Cuitiño et al., 2019; Cura Costa et al., 2021; Hecht et al., 2022). FUCCI has also proven instrumental in cancer research through the delineation of cell cycle dependent responses (Ganem et al., 2014; Krenning et al., 2014; Ryl et al., 2017; Molinie et al., 2019; Nano et al., 2019; Rajal et al., 2021; Wang et al., 2021; Gemble et al., 2022; Kozyrska et al., 2022; Zeng et al., 2023).

Apart from timelapse imaging, the FUCCI intensity-based fluorescence readout is compatible with fluorescence-activated cell sorting (FACS). By setting intensity thresholds to select the co-occurrence of hCdt1 (30/120) in G1 and hGem (1/110) that indicates S/G2/M phases, a highly synchronous G1/S cell population can be enriched (Feringa et al., 2016). Similar approaches were employed to enable cell cycle synchronised transcriptome and proteomes analysis to be performed on either bulk sorted or single-cells (Boström et al., 2017; Herr et al., 2020; Hsiao et al., 2020; Mahdessian et al., 2021; Krenning et al., 2022).

## Limitations of the original FUCCI system

Despite its useful capabilities, the FUCCI system does have limitations that restrict its use for specific questions. One key limitation is its inability to tell the difference between the S phase and the G2 phase (Sakaue-Sawano et al., 2008). Since Geminin remains stable throughout both phases, cells in S and G2 fluoresce similarly, making it difficult to distinguish DNA replication from G2. This lack of phase distinction complicates research that requires accurate mapping of DNA synthesis, repair, or pre-mitotic surveillance mechanisms. Furthermore, FUCCI cannot distinguish between G0 and G1 phases, as both are characterised by Cdt1 accumulation and Geminin degradation. This limitation reduces the system’s usefulness in studies focused on cell quiescence, requiring the use of additional reporters to differentiate G0 and G1 populations (Henderson et al., 2014; Oki et al., 2014).

Furthermore, the original FUCCI reporters were designed based on human degradation motifs and thus depend on SCF^Skp2^ and APC^Cdh1^ activity (Wohlschlegel et al., 2000; Nishitani et al., 2001; Li et al., 2003; Vodermaier, 2004). However, in model organisms like zebrafish and *Drosophila*, these degradation pathways differ significantly. For example, zebrafish cells primarily utilise CUL4^Ddb1^-mediated degradation of Cdt1, rendering the human-derived FUCCI constructs ineffective (Sugiyama et al., 2009). Likewise, *Drosophila* requires species-adapted degrons to accurately track phases which led to the development of species specific FUCCI variants (Zielke et al., 2014).

Another technical limitation stems from the variability caused by the method of reporter delivery. Lentiviral transduction, often used to generate stable FUCCI-expressing lines, results in random genomic integration, which leads to varied expression levels (Formas-Oliveira et al., 2025). Although this variability can be reduced through single-cell cloning or fluorescence-based sorting, these procedures add complexity and duration to experimental workflows and may not fully prevent expression instability in long-term studies.

## Advancements in FUCCI design

To overcome the limitations of the original FUCCI design, several improved variants have been developed. FUCCI2 enhances the original by incorporating brighter fluorescent proteins such as mCherry and mVenus to increase signal strength and enable imaging in deep tissues or low expression environments (Abe et al., 2013). PIP-FUCCI employs the PIP degron of Cdt1 (1–17) to replace Cdt1 (30–120), to delineate G1 and G2/M to improve the precision of cell cycle phase reporting (Grant et al., 2018). FUCCI4 expands the system further by adding fluorescently tagged linker histone H1.0 to detect M phase and the SLBP(18–126) to delineate S to G2 transition (Bajar et al., 2016). In combination with Cdt1 (30–120) and Geminin (1–110), all four main cell cycle phases (G1, S, G2, and M) can be identified in FUCCI4, allowing for more detailed temporal tracking of cell cycle progression (Bajar et al., 2016).

Species-specific adaptations have also broadened the application of FUCCI in non-mammalian models. zFUCCI incorporates zebrafish-specific degrons, allowing for precise phase tracking in zebrafish embryos, while Fly-FUCCI is designed to match *Drosophila*’s unique degradation pathways (Sugiyama et al., 2009; Zielke et al., 2014). These adaptations have been vital for utilising FUCCI-based analysis in developmental biology and tissue regeneration across various organisms.

A particularly notable advancement is FUCCI(CA), which incorporates CUL4^Ddb1^ sensitive degrons into the Cdt1 reporter and the replacement of the conserved RRL motif to AAA to prevent SCF^Skp2^ dependent degradation (Sakaue-Sawano et al., 2017). This variant allows for better resolution of G1, S, and G2 phases in mammalian systems since the mutant hCdt1 peptide is only actively degraded by Cul4^Ddb1^ in S-phase. FUCCI(CA) has proven especially useful in analysing interphase regulation, providing insights into phase-specific DNA damage responses and checkpoint control mechanisms (Knoblochova et al., 2023; Szmyd et al., 2025).

In summary, the FUCCI reporter system is a versatile cell cycle reporter which is easily employed in a variety of biological systems. However, since FUCCI relies on cell cycle-specific degradation of either Cdt1 or Geminin to infer cell cycle phase, it does not directly monitor functional processes such as DNA synthesis in S phase. Due to this limitation, alternative cell cycle reporter systems were subsequently developed and will be discussed in the following sections.

## Kinase translocation reporters (KTRs)

Kinase translocation reporters (KTRs) are genetically encoded biosensors that can detect kinase activity in real time by converting phosphorylation events into spatial changes in subcellular localisation (Hahn et al., 2009; Regot et al., 2014; Kudo et al., 2017). Unlike phase-specific reporters such as FUCCI that infer cell cycle phase by detecting cell cycle specific protein degradation, KTRs infer cell cycle status indirectly by measuring the kinase activities of cell cycle regulators such as Cyclin Dependent Kinases (CDKs). As kinase activities of CDKs are known to drive cell cycle transitions, the oscillations in the kinase activity provides a useful indicator of upstream signalling events that promote cell cycle phase transitions (Coudreuse and Nurse, 2010).

A typical KTR consists of a kinase-specific substrate motif flanked by a nuclear localisation signal (NLS), a nuclear export signal (NES), and a fluorescent reporter protein (Gu et al., 2004; Regot et al., 2014; Maryu et al., 2016). In the absence of phosphorylation, the reporter localises to the nucleus due to dominant NLS activity. Upon phosphorylation by a specific kinase, the negative charge introduced disrupts nuclear import and enhances NES-mediated export, causing the reporter to accumulate in the cytoplasm. This process is reversible as dephosphorylation restores its nuclear localisation. By measuring the ratio of fluorescence intensity between the cytoplasm and nucleus, the dynamics of kinase activity can be inferred with high temporal resolution.

The Human DNA Helicase B (HDHB)-based CDK2 reporter is a widely used examples of KTRs in cell cycle research (Figure 1B). HDHB is regulated by phosphorylation at multiple CDK target sites, most notably serine 967 in its C-terminal region, which causes the shuttling of the HDHB fragment between the nucleus and cytoplasm in response to CDK2 activity (Gu et al., 2004; Hahn et al., 2009). The HDHB biosensor was originally created by fusing the C-terminal peptide of HDHB to a red fluorescent protein (tdimer2) and expressing it under a CMV promoter (Hahn et al., 2009). Since then, several variants have been developed by fusing the HDHB fragment to brighter fluorescent markers such as mVenus and mTurquoise2 (Spencer et al., 2013; Hoffman et al., 2025).

KTRs have proven especially useful in visualising kinase dynamics related to cell cycle regulation, commitment, and checkpoint control. The HDHB-based CDK2 reporter has provided critical insight into the mechanism through which CDK2 activity at mitotic exit determines whether a cell resumes proliferation or transitions into a transient G0-like state (Cappell et al., 2016; Cappell et al., 2018; Yang et al., 2017; Min et al., 2020). Additionally, the translocation of the HDHB-based CKD2 KTR is highly responsive to rapid changes in CDK activity, with measurable changes in the cytoplasm/nucleus ratio within minutes of CDK2 inhibition (Spencer et al., 2013).

Beyond CDK2, several KTRs have been developed for other key cell cycle-regulating kinases. Among them is a CDK4/6-specific reporter used to study the sequential activation cascade between both CDK4/6 and CDK2 in governing the G1/S transition (Kim et al., 2022). Using kinase-specific KTRs, it was demonstrated that CDK4/6 activity is sufficient to initiate Rb phosphorylation and E2F activation, but CDK2 activity is necessary for S phase commitment (Kim et al., 2022).

KTRs provide highly quantitative and sensitive readouts of kinase activities, and pair well with single cell fluorescent imaging studies. By measuring kinase activities and computationally aligning these signalling events to cellular dynamics, we can accurately associate cellular events to changes in kinase activities *in vivo*. This approach have been used extensively to study multifunctional kinases that are influenced by the cell cycle to regulate proliferative or cell differentiative responses (Regot et al., 2014; Pokrass et al., 2020; Hanson and Batchelor, 2022; Kim et al., 2023). For example, using ERK KTRs researchers were able to demonstrate that low ERK activity after mitotic exit correlates with NANOG stabilisation and maintenance of stem cell pluripotency (Pokrass et al., 2020). Similar results were obtained in *C. elegans*, where ERK KTRs were employed to demonstrate the role of ERK in cell fate specification (de la Cova et al., 2017). These studies demonstrate the versatility of KTRs in studying signalling events in the cell cycle that influences cell differentiation.

Another major advantage of KTRs is their modularity. Fluorescent tags can be easily swapped to enable multiplexed imaging with minimal spectral overlap (Kudo et al., 2017). Multiplexed KTR systems for ERK, JNK, and p38 have been used to simultaneously monitor cellular responses to stress and DNA damage in real time (Hanson and Batchelor, 2022). More recently, p38 and JNK KTRs were used to measure activation kinetics in response to ultraviolet light at single-cell level (Sinha et al., 2024). These studies demonstrate the feasibility of combining multiple KTRs to reveal insights on multiple signalling pathways that the cell cycle responds to during cellular stress.

## Limitations and considerations of KTRs

Although KTRs provide valuable dynamic readouts, several limitations may restrict their application. Accurate signal quantification depends on clear separation of nuclear and cytoplasmic regions, requiring high-resolution imaging and reliable nuclear markers. In cell types with irregular shapes, tracking nucleocytoplasmic translocation precisely becomes difficult. Additionally, nuclear envelope ruptures, occurring in some cancer cells, senescent cells, or following mitotic errors, permit passive diffusion of KTRs, which interferes with localisation-based signal analysis (Maciejowski and Hatch, 2020; Kamikawa et al., 2023). Furthermore, since the nuclear transport of KTRs depends on the nuclear transport machinery of the cell, it is possible that KTRs developed from mammalian systems may not function in evolutionary divergent organisms due to differences in the nuclear transport machinery. Therefore, choosing suitable cell models with stable nuclear structures is crucial for dependable KTR imaging.

KTR specificity can also be affected by kinase crosstalk. It was recently reported that CDK2 can phosphorylate ERK and p38 KTRs, leading to false-positive translocation events (Hoffman et al., 2025). This emphasises the importance of carefully selecting KTR peptide sequences and including suitable experimental controls to verify reporter specificity. If crosstalk is identified, alternative peptide sequences or mutating nonspecific phosphorylation sites to enhance target specificity should be considered (Creixell et al., 2015; Miller and Turk, 2018). Alternatively, employing appropriate drug controls that block kinase crosstalk to demonstrate reporter specificity can be implemented (Hoffman et al., 2025).

Furthermore, while KTRs provide a real-time readout of kinase activity, these signals are generally presented across a continuous spectrum. For instance, CDK2 activity gradually increases during S and G2, making it difficult to accurately differentiate between these phases based solely on the KTR signal (Spencer et al., 2013; Cappell et al., 2016). This requires an additional fluorescent reporter to accurately indicate the entry into S-phase cell cycle phase (Cappell et al., 2016).

In summary, kinase translocation reporters occupy a unique niche among fluorescent cell cycle indicators. Their principal advantage lies in providing a functional readout of kinase signalling dynamics that drive or accompany cell cycle progression. In contrast to systems like FUCCI, which report on degradation-based phase transitions, which reflect gene expression dynamics, KTRs measure the activity of key upstream regulators in real time. This makes KTRs particularly powerful for studying rapid signalling responses, phase bifurcation events, and stress-induced alterations in cell cycle control.

## DNA replication foci based cell cycle reporters

DNA replication foci–based reporters utilise the spatial and temporal organisation of DNA replication machinery to monitor S-phase progression. These systems typically employ fluorescently tagged proteins that associate with replication forks, such as proliferating cell nuclear antigen (PCNA) (Figure 1C). PCNA is a conserved protein that forms a ring-like structure around DNA at replication forks, bringing together proteins necessary for DNA synthesis (Moldovan et al., 2007; Mailand et al., 2013). Because PCNA displays distinct subnuclear localisation patterns during the cell cycle, it has been adopted as a fluorescent reporter for monitoring DNA replication dynamics in live cells (Zerjatke et al., 2017). By tracking the formation and dissolution of replication foci in live cells, researchers can delineate the onset, progression, and completion of DNA replication in real time (Leonhardt et al., 2000). These reporters offer unparalleled resolution of S-phase dynamics and have become essential tools in studies of replication timing, genome stability, and the cellular response to replication stress (Essers et al., 2005; Chagin et al., 2016; Chao et al., 2017).

Early fluorescent PCNA reporters involved fusing PCNA to green fluorescent protein (GFP) (Leonhardt et al., 2000). When expressed under a CMV promoter in C2C12 cells, GFP-PCNA formed characteristic replication foci during S phase, enabling direct visualisation of DNA synthesis. Building upon this, a dual-reporter cell line stably expressing EGFP-PCNA and histone H2B-mCherry in HeLa cells was developed, which allowed simultaneous observation of replication foci and chromatin condensation (Piwko et al., 2010). Additionally, other exogenous promoters such as the PGK promoter (Chao et al., 2017) and endogenous knock-in strategies, where native PCNA was fused with mRuby in hTERT RPE-1 cells (Zerjatke et al., 2017), were created. These approaches provided reliable cell cycle phase classification by faithfully recapitulating PCNA dynamics, demonstrating versatility in reporter design and expression strategies for accurate live-cell cell cycle monitoring.

The use of PCNA-based reporters provides several key benefits. The quick and reversible shift of PCNA between a diffuse nuclear presence and replication foci offers a highly sensitive and precisely timed marker of S phase progression. As the determination of S phase entry is dependent on the spatial redistribution of PCNA from a diffused state into a punctate state, changes in fluorescent intensity due to photobleaching would have a limited impact on the reliability of PCNA-based reporters in determining the cell cycle stage. This feature provides a distinct advantage over other fluorescent reporters that depend on changes in expression levels, such as FUCCI, since photobleaching is a common problem in long term imaging experiments. Furthermore, cell-permeable fluorescently labelled nanobodies that recognise endogenous PCNA have been developed, allowing the visualisation of PCNA dynamics in live cells, bypassing the need to generate stable fluorescent cell lines (Schneider et al., 2021).

## Limitations and considerations of employing fluorescent PCNA reporters

Despite their strengths, PCNA reporters also have limitations that warrant careful consideration. Early overexpression experiments of full-length PCNA have reported that PCNA overexpression impacts cell cycle progression. Studies have shown that increases in PCNA expression lead to increased replication stress, disruption of growth control and could contribute to malignant cell transformation (Fukami-Kobayashi and Mitsui, 1999; Johnson et al., 2016). Mechanistically, this could be due to the sequestration of proteins from their regular sites of action, as PCNA is an important binding partner for many cell cycle proteins, including p21 (Prives and Gottifredi, 2008; Boehm et al., 2016). Thus, it is important to use less disruptive methods like endogenous fluorescently tagged PCNA or fluorescently labelled PCNA nanobodies to visualise the DNA replication foci as they are less likely to perturb the natural dynamics of cell cycle progression.

The functional role of PCNA is not limited to DNA replication. It also functions in DNA repair processes. In response to genotoxic stress, PCNA can form foci outside of S phase to facilitate DNA repair (Balajee and Geard, 2001). Conversely, events that stall the progression of the DNA replication machinery, such as exposure to aphidicolin, would limit the formation of PCNA foci (Chen et al., 2025). These issues can complicate the interpretation of replication-specific signals. Therefore, when using fluorescent PCNA, it's important to avoid using PCNA-based reporters in cell lines experiencing elevated replication stress and set reasonable thresholds for the number of PCNA foci used to identify S-phase, since DNA repair can also cause foci to form outside of S-phase (Chao et al., 2017). Furthermore, the changes in fluorescent PCNA intensity as cells progress from G1 to G2 are a poor indicator of cell cycle progression (Zerjatke et al., 2017). This necessitates generating single-cell lineages of fluorescently tagged PCNA cell lines from time-lapse images before accurate cell cycle boundaries can be determined.

In addition, PCNA’s dynamic localisation patterns from diffuse to punctate foci pose challenges for foci detection and nuclear segmentation in image analysis. To reliably detect PCNA foci, confocal microscopes, such as spinning disk-based microscopes, are preferred. This is because widefield microscopes at lower magnifications (below 20x) may lack the necessary resolution to reliably detect the formation of DNA replication foci, especially during early S phase. When PCNA fluorescence is used as the sole nuclear marker, accurate delineation of nuclear boundaries can be difficult using conventional segmentation approaches (Piwko et al., 2010). This necessitates the use of an additional fluorescent marker for the nucleus to enable the reliable segmentation and tracking of cells (Piwko et al., 2010; Zerjatke et al., 2017). The need to include a fluorescently tagged nuclear marker may be a disadvantage in multiplex experiments since adding additional fluorescent channels may increase phototoxicity. However, recent advances in deep learning based image analysis have overcome this limitation, providing improved segmentation accuracies to recognise PCNA’s characteristic fluorescence patterns, enabling the automated segmentation, cell cycle classification and single cell lineage tracking without the need for a fluorescent nuclear marker (Gui et al., 2022).

In summary, PCNA-based reporters occupy a vital role within the fluorescent cell cycle reporter toolkit, particularly for detailed and dynamic studies of DNA replication during S phase. Their physiological relevance and temporal sensitivity make PCNA reporters valuable for investigating replication timing and replication stress responses.

## Future perspectives

In this review, we have summarised the commonly used classes of fluorescent cell cycle reporters, each capturing distinct but complementary aspects of cell cycle progression (Table 1; Figure 1). Despite their usefulness, individual cell cycle reporters may be insufficient in capturing the full complexity of cell cycle dynamics, especially during conditions of cellular stress.

**TABLE 1:** Brief summary of the different of cell cycle reporters.

Reporter system	Mechanistic basis	Strengths	Weaknesses
FUCCI (Original and variants)	Detects the change in cell cycle specific ubiquitin-mediated proteolysis of proteins	Simple phase resolution; applicable across organisms; suitable for long-term and *in vivo* imaging; compatible with FACS.	Original FUCCI cannot distinguish S from G2; cannot separate G0 from G1; species-specific adaptations often required; expression variability
Kinase Translocation Reporters (KTRs)	Detects the nuclear and cytoplasmic presence of phosphorylated peptide sequences to read out kinase activities	Direct functional readout of kinase activity; high temporal resolution; modular and multiplexable; reveals regulatory transitions	Requires clear nuclear/cytoplasmic segmentation; sensitive to nuclear envelope integrity; kinase crosstalk can cause false signals
DNA Replication Foci (PCNA-based)	Detects the dynamic relocalisation of fluorescently tagged PCNA to form replication foci during DNA synthesis, reflecting S-phase dynamics	High sensitivity and precision for S-phase entry/progression; reversible and robust readout; less affected by expression fluctuations	PCNA also marks DNA repair, complicating S-phase identification; nuclear markers should be employed to help segment the nucleus

It has also been pointed out in several studies that, depending on the biological properties measured, different reporters can give conflicting results of cell cycle phases. For instance, the DNA replication foci-based PCNA and CDK2 KTR-specific HDHB reporters may yield inconsistent outcomes during replication stress or DNA damage induction (Essers et al., 2005; Daigh et al., 2018). Under such conditions, PCNA foci formation might be disrupted in S phase (Essers et al., 2005), and cytoplasmic HDHB localisation may decrease transiently during S phase (Daigh et al., 2018), leading to possible misclassification of cell cycle phases when relying on only one reporter. This highlights the limitations of single-reporter methods, especially in genomically unstable cancer cell models experiencing high levels of replication stress (Burrell et al., 2013; Hanahan, 2022).

Combining multiple fluorescent reporters offers an advantage in understanding complex regulatory mechanisms that single reporters cannot fully resolve. For example, integrating CDK2 activity sensor with components of either Cdt1 or Geminin fragments in the FUCCI reporter enables the accurate determination of the G1/S transition (Cappell et al., 2016; Ratnayeke et al., 2023). This multi-reporter combination was instrumental in determining the molecular roles of various cell cycle regulators in mediating G1/S transition (Spencer et al., 2013; Cappell et al., 2016; Cappell et al., 2018; Daigh et al., 2018; Chung et al., 2019; Liu et al., 2020; Nathans et al., 2021; Ratnayeke et al., 2023). The multi-reporter approach can also be used to investigate how intercellular signalling by various kinases impacts cell cycle transitions using a combination of KTR based reporters. In a recent study, an array of reporters, including a novel G2-specific kinase translocation reporter (KTR), was used to identify a p53-independent mechanism of stress-induced G2 exit, mediated by SAPK signalling and early activation of APC/C-Cdh1 (McKenney et al., 2024). These findings underline that using multi-reporter strategies not only improves temporal and spatial resolution of cell cycle transitions but also reduces misinterpretations caused by changes in marker behaviour. Furthermore, these studies highlight the advantages of combining different cell cycle biosensors as they can help dissect cell cycle transitions and stress responses with unprecedented detail.

## Conclusion

Genetically encoded fluorescent cell cycle reporters offer a powerful toolkit for accurately studying cell cycle dynamics in live cells. However, as discussed in this review, careful consideration must be given to selecting the appropriate reporter. The choice of reporter should depend on the biological experiment as some of the reporters may be suboptimal or unsuitable. Recent demonstrations of multi-reporter strategies expand the utility of these cell cycle reporters. By carefully selecting the best reporter and employing multi-reporter strategies that detect distinct and complementary aspects of cell cycle progression, researchers can overcome the limitations of single reporters. Together, these reporters are not only transforming the way we study the cell cycle but also unlocking new insights into the fundamental processes that govern cell fate and function.
